# Angiotensin II type 2 receptor promotes apoptosis and inhibits angiogenesis in bladder cancer

**DOI:** 10.1186/s13046-017-0542-0

**Published:** 2017-06-09

**Authors:** Nana Pei, Yingying Mao, Pengfei Wan, Xinglu Chen, Andrew Li, Huiying Chen, Jinlong Li, Renqiang Wan, Yanling Zhang, Hongyan Du, Baihong Chen, Guangyu Jiang, Minghan Xia, Colin Sumners, Guixue Hu, Dongsheng Gu, Hongwei Li

**Affiliations:** 1 Department of Clinical Pathology, The First Affiliated Hospital of Jinan University, Guangzhou, Guangdong China; 2 School of Laboratory Medicine and Biotechnology, Southern Medical University, 1023 South Shatai Road, Guangzhou, Guangdong 510515 China; 3 Department of Biomedical Engineering, The Johns University School of Medicine, Baltimore, USA; 4 Department of Otolaryngology-Head and Neck Surgery, Guangdong No. 2 Provincial People’s Hospital, Guangzhou, Guangdong China; 5 Department of Physiology and Functional Genomics, University of Florida, Gainesville, FL USA; 6 College of Animal Science and Technology, Jilin Agricultural University, Xincheng Street No. 2888, Changchun, 130118 People’s Republic of China; 7 Department of Urology, the 421 St Hospital of PLA, No. 350, Xinggang Rd, Haizhu district, Guangzhou, Guangdong 510318 China

## Abstract

**Background:**

Bladder cancer (BCa) is the ninth most common form of cancer in the world. There is a continuing need not only for improving the accuracy of diagnostic markers but also for the development of new treatment strategies. Recent studies have shown that the renin-angiotensin system (RAS), which include the angiotensin type 1 (AT1R), type 2(AT2R), and Mas receptors, play an important role in tumorigenesis and may guide us in meeting those needs.

**Results:**

In this study, we first observed that AT1R and Mas expression levels were significantly upregulated in BCa specimens while AT2R was significantly downregulated. Viral vector mediated overexpression of AT2R induced apoptosis and dramatically suppressed BCa cell proliferation in vitro, suggesting a therapeutic effect. Investigation into the mechanism revealed that the overexpression of AT2R increases the expression levels of caspase-3, caspase-8, and p38 and decreases the expression level of pErk. AT2R overexpression also leads to upregulation of 2 apoptosis-related genes (BCL2A1, TNFSF25) and downregulation of 8 apoptosis-related genes (CASP 6, CASP 9, DFFA, IGF1R, PYCARD, TNF, TNFRSF21, TNFSF10, NAIP) in transduced EJ cells as determined by PCR Array analysis. In vivo, we observed that AT2R overexpression caused significant reduction in xenograft tumors sizes by downregulation VEGF and induction of apoptosis.

**Conclusions:**

Taken together, the data suggest that AT1R, AT2R or Mas could be used as a diagnostic marker of BCa and AT2R is a promising novel target gene for BCa gene therapy.

## Background

Bladder cancer (BCa) is a cancer of the genitourinary system that typically originates from cells that line the inside of the bladder [1]. Although BCa can occur at any age, it is typically seen in older adults and in high risk individuals with a history of smoking and occupational exposure to carcinogens [2, 3]. Roughly 70% of BCa cases are non-invasive bladder cancer that can be successfully physically excised, however, the other cases have a risk of progression to muscle invasive bladder cancer and metastasis to distant organs, endangering of the lives of patients. Despite improvements in therapy due to advances in diagnostic and surgical techniques, the majority of deaths caused by BCa result from metastasis that are resistant to conventional therapy [4–6]. Thus, novel treatment strategies for bladder cancer are urgently needed.

The renin-angiotensin system (RAS) consists of renin, angiotensinogen, angiotensin-converting enzyme, and multiple angiotensin peptides. A major regulatory component is angiotensin II (Ang II) which acts through the angiotensin type 1 (AT1R) and type 2 (AT2R) receptors and has been suspected of playing a major role in carcinogenesis [7–10]. In contrast to the well-known harmful activities of AT1R, AT2R is considered to be the protective arm of RAS and often acts in opposition to AT1R [11]. Studies have shown that AT1R antagonists prevent angiogenesis and growth of xenograft models of human BCa [12, 13]. AT2R is known to inhibit cell proliferation and stimulate apoptosis in a variety of cell lines including vascular smooth muscle cells, cardiomyocytes, endothelial cells, prostate cancer cells, and lung cancer cells [14–19]. These findings suggest AT2R as a potential cancer therapeutic and no evidence for AT2R effectiveness in BCa has been documented until now.

In this study, we investigated the therapeutic potential of AT2R in BCa using adenovirus vectors. We first confirmed the role of adenoviral-induced AT2R overexpression on inhibiting proliferation and inducing apoptosis in bladder carcinoma cells. Second, we investigated the role of AT2R overexpression on BCa tumorigenesis in a xenograft murine model. Finally, we explored the mechanism of AT2R on BCa in vitro. This study demonstrates AT2R as a potential therapeutic agent for BCa and may allow us to gain further insight into BCa pathogenesis.

## Methods

### Cell cultures

Human bladder cancer cell lines (EJ, UM-UC-3, 5637) were obtained from the American Type Culture Collection (Rockville, MD) and were cultured in RPMI-1640 (Invitrogen) medium supplemented with 10% FBS under 5.0% CO_2_. Sera and media were purchased from Invitrogen and American Type Culture Collection. HEK 293A cells were cultured in Dulbecco’s modified Eagle’s medium (DMEM; Invitrogen).

### Clinical specimens

Primary bladder cancer biopsy specimens and normal biopsies were obtained from Nanfang Hospital (Guangzhou, Guangdong, China). The clinical information of patients was previously described [20]. Both tumor and normal tissues were histologically confirmed by H&E (hematoxylin and eosin) staining. Informed consent was obtained from each patient, and the research protocols were approved by the Ethics Committee of Nanfang Hospital.

### Recombinant adenoviral construction and preparation

Recombinant adenoviral vectors were constructed, prepared, and titrated as previously described [21]. These vectors were: an adenoviral vector containing the enhanced green fluorescent protein gene controlled by a cytomegalovirus promoter (Ad-CMV-eGFP) and an adenoviral vector containing genomic AT2R (G-AT2R) DNA with introns 1 and 2 and the encoding region and enhanced green fluorescent protein gene controlled by cytomegalovirus promoters (Ad-G-AT2R-eGFP).

### Cell transduction

For viral transduction, bladder cancer cell line cells (5 × 10^5^) were seeded into six-well Nunc tissue culture plates. On the following day, cells were transduced with Ad-G-AT2R-eGFP or the control vector Ad-CMV-eGFP and changes in cell morphology were observed using an Olympus IX71 fluorescence microscope (Olympus America Inc., PA, USA). Transduced cells were used 24 to 48 h later, depending on the specific protocol.

### AT2R immunostaining

Cells transduced with Ad-G-AT2R-eGFP or Ad-CMV-eGFP for 48 h were washed briefly with Dulbecco’s PBS and then fixed for 10 min at 4 °C with cold methanol. Immunostaining was then done on the fixed cells as detailed previously [22] using a goat anti-AT2R receptor polyclonal antibody (1:200; Santa Cruz Biotechnology) followed by Alexa Fluor 594 goat anti-rabbit IgG (1:1,000; Invitrogen) as the secondary antibody. AT2R immunoreactivity (red) and green fluorescence (from eGFP) were detected using an Olympus BX41 fluorescence microscope.

### Cell proliferation and cytotoxicity assays

Cell proliferation and cytotoxicity were evaluated using a CytoScan WST-1 Cell Proliferation Assay (G-Biosciences). WST-1 reagent was added to the culture medium (1:10 dilution), and absorbance was measured at 450 nm.

### Apoptosis assays

Apoptosis of viral vector-transduced cells was measured using a DeadEnd Colorimetric terminal deoxynucleotidyl transferase-mediated dUTP nick end labeling (TUNEL) System (Promega) and One Step TUNEL Apoptosis Assay Kit (Beyotime) as described previously [17]. The number of stained cells that exhibit apoptotic-like morphology was assessed by counting cells from 10 randomly chosen fields per well.

### Caspase-3-like protease activity

Caspase-3–like protease activity was assessed using the BD ApoAlert caspase-3 colorimetric assay kit (BD Biosciences) as described by the manufacturer. Transduced and control cells (10^6^) were lysed in the lysis buffer contained in the kit followed by centrifugation (15,000 × g for 10 min at 4 °C). Caspase-3–like activity was assessed in supernatants by following the proteolytic cleavage of the colorimetric substrate Ac-DEVD-pNA. Samples were read at 405 nm in a spectrophotometer using a 100 μL quartz cuvette. DEVD-z-DEVD-fmk, a specific inhibitor of caspase-3, was used to confirm assay specificity.

### RNA isolation, reverse transcription, and quantitative real-time RT-PCR

Total RNA was extracted using an RNeasy Mini-Kit (Qiagen) according to the manufacturer’s instructions. Quantitative real-time RT-PCR was performed on an ABI 7500 real-time PCR system (Applied Biosystems) as described previously [23]. The primers are listed in Table 1. The samples were quantified by the comparative △△C_T_ method by using human GAPDH as the internal standard.

### Apoptotic gene expression analysis using real-time PCR array

Genes involved in apoptosis were performed by means of the Human Apoptosis RT^2^ Profiler PCR Array (PAHS-012Z; *SAB*iosciences, USA) as described previously [23]. Genes with relative fold changes greater than ± 2 were considered as up or downregulated in expression. Genes that yielded a *p*-value of <0.05 were considered to display statistical significance for the study.

### Western blot analysis

Western immunoblots were run as described previously [24]. Primary antibodies and their sources were as follows. Anti-total p38 MAPK, anti-phosphorylated p38 (pp38) MAPK, anti-Erk, anti-phosphorylated Erk, anti-activated caspase 3, anti-activated caspase 8 were from Cell Signaling Technology. Anti-*β-*actin and the secondary antibodies horseradish peroxidase-conjugated anti-rabbit IgG and anti-rabbit IgG were from Sigma-Aldrich. Anti-goat IgG was from Santa Cruz Biotechnology.

### Tumor growth assay

Female BALB/c nude mice aged 4 to 5 weeks were purchased from the Institute of Comparative Medicine and Center of Laboratory Animals of the Southern Medical University (SMU). Animal handling and experimental procedures were approved by the Animal Experimental Ethics Committee of SMU. Athymic mice were subjected to s.c. injections of human EJ bladder cancer cells (1.0 × 10^6^) in Matrigel (50:50) into the lower flank to induce tumor growth. After the tumors reached ∼ 50 mm^3^, the mice were placed into three groups at random. The animals received intratumor injections of Ad-G-AT2R-eGFP (1 × 10^9^ vg/mouse), Ad-CMV-eGFP (1 × 10^9^ vg/mouse) or PBS with multiple target points after measuring the tumor size every 3 days. Each group contained 6 mice and the experiment was repeated 3 times. Intratumor injections were conducted a total of 9 times. Before each injection, the length and width of the tumors were measured by using a Vernier caliper. Following the study the mice were anesthetized and euthanized by decapitation and tumors were dissected. To confirm the transgene expression within the tumor, a series of 7-μM-thick fresh-frozen sections of the samples were made using a microtome at low-temperature and observed under fluorescence microscopy. Tumor volumes were calculated as follows: volume = (D × d^2^)/2, where D meant the longest diameter and d meant the shortest diameter. Tumors were isolated for Western blot and quantitative real-time RT-PCR analysis, or fixed in 10% buffered formalin and used for histologic and immunohistochemical analysis.

### Immunohistochemistry

Tumors were fixed in 4% paraformaldehyde for 24 h and incubated in 70% ethanol for 48 h before embedding in paraffin. The embedded tumors were cut into 5-μm-thick sections and stained with H&E to determine morphology. Cell proliferation in the transplanted tumors was analyzed for Ki67 (1:200; Abcam) expression. Apoptosis of viral vector transduced cells in tumors was also measured using a DeadEnd Colorimetric terminal deoxynucleotidyl transferase–mediated dUTP nick end labeling (TUNEL) System (1:100; Promega). Visualization was achieved using the EnVision + peroxidase system (Dako). Ki67 immunoreactive cells were expressed as a percentage of the total cell number of examined fields. The apoptosis was assessed by counting the number of the apoptotic cells from 10 random fields per tumor. Counts were done by an individual who was blinded as to the treatment.

### Statistical analysis

SPSS 21.0 software was used for statistical analysis. Data are presented as mean ± standard deviation (SD) from 3 to 6 independent experiments. Statistical differences were evaluated by one-way ANOVA followed by Dunnett’s post hoc test. The criterion for statistical significance was set at *P* < 0.05.

## Results

### AT1R, AT2R and Mas expression in human BCa clinical specimens

**Fig. 1:**
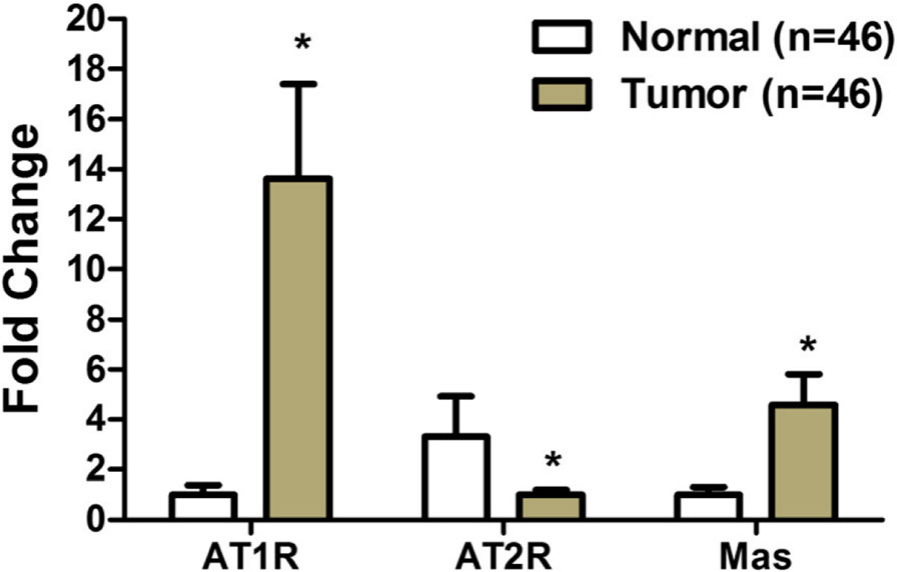
Detection of AT1R, AT2R and Mas mRNA expression in BCa clinical specimens. Expression of AT1R, AT2R and Mas in BCa samples and their corresponding para-carcinoma samples from 46 patients. AT1R, AT2R and Mas abundance was normalized to GAPDH. * *P* < 0.01 vs. normal tissues

We examined the mRNA expression levels of AT1R, AT2R and Mas in BCa samples and their corresponding para-carcinoma samples from 46 patients. The data showed that the average expression level of AT1R and Mas were increased by about 14- and 4-fold, respectively (Fig. 1; *P* < 0.01), and AT2R was reduced by approximately 3-fold in BCa specimens than in control tissues (Fig. 1; *P* < 0.01). The increased or decreased levels of these receptor mRNAs suggest that AT1R, AT2R or Mas may serve as a diagnostic marker in bladder cancer.

### Endogenous and adenoviral-mediated expression of AT2R in bladder cancer cells

The presence of endogenous AT2R in human bladder cancer cell lines was first assessed using real-time RT-PCR. Endogenous AT2R mRNA expression was minimally detectable in basal bladder cancer cell lines. Ct values for endogenous AT2R mRNA in untreated EJ, UM-UC-3 or 5637 cells were all greater than 35.0, which are defined as within the negative range.

**Fig. 2:**
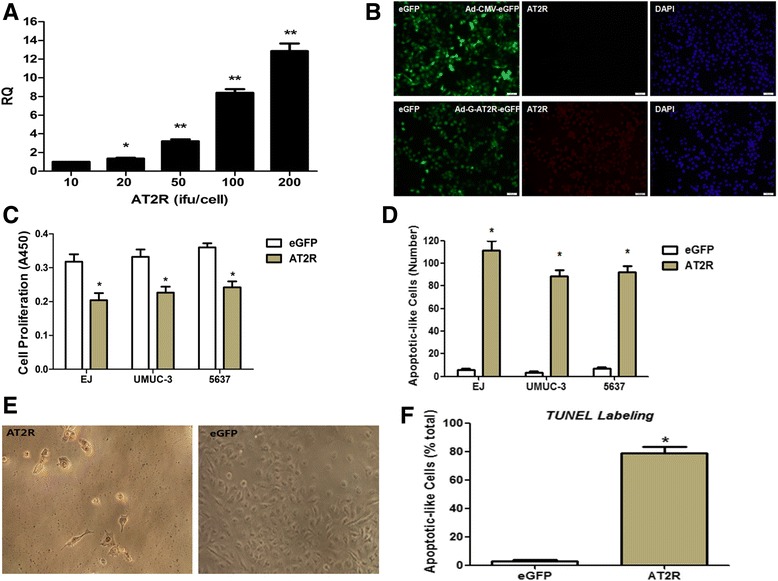
Increased expression of AT2R in BCa cells: effects on cell proliferation and apoptosis. **a** AT2R mRNA expression in EJ cells following transduction with gradient doses of Ad-G-AT2R-eGFP (10 ifu, 20ifu, 50 ifu, 100 ifu, 200 ifu/cell). Columns, mean (*n* = 3); *P < 0.05 and ** *P* < 0.01 *vs* cells transduced with 10 ifu of Ad-G-AT2R-eGFP. **b** EJ cells were transduced with either Ad-CMV-eGFP or Ad-G-AT2R-eGFP (100 ifu/cell) for 24 h as described in Materials and Methods. Incubations were followed by detection of eGFP fluorescence and AT2R immunoreactivity using an anti-AT2R antibody. Representative fluorescence micrographs from Ad-CMV-eGFP–transduced and Ad-G-AT2R-eGFP–transduced cells, showing eGFP fluorescence, AT2R immunostaining and DAPI in each treatment condition. Scale bars, 50 μm. **c** BCa cells underwent transduction with either Ad-CMV-eGFP or Ad-G-AT2R-eGFP (100 ifu/cell) for 24 h. Following this, WST-1 reagent was added to the culture medium (1:10 dilution), and absorbance was measured at 450 nm. Columns, mean A450 nm from three experiments; bars, SD. * *P* < 0.01 *vs* Ad-CMV-eGFP–transduced cells. **d** BCa cells were transduced with either Ad-CMV-eGFP or Ad-G-AT2R-eGFP (100 ifu/cell) for 2 d as described in the Methods. Incubations were followed by detection of eGFP fluorescence and counting of cells that display apoptotic-like morphology from 10 random fields per well. Columns, mean from three separate experiments; bars, SD. * *P* < 0.01 *vs* corresponding eGFP-transduced cells. **e** EJ cells were transduced with either Ad-CMV-eGFP or Ad-G-AT2R-eGFP (100 ifu/cell) for 2 d, and apoptotic cells were detected using the DeadEnd Colorimetric TUNEL System kit. A representative phase-contrast micrograph was shown from each treatment condition. The *brown-colored* nuclei represent TUNEL-positive (apoptotic) cells. **f** quantification of the TUNEL-positive cells as a percent of the total number of cells in the dish. Columns, mean of three experiments; bars, SE. * *P* <0.001 *vs* eGFP group

EJ cells were then transduced with either increasing doses of Ad-G-AT2R-eGFP or the control vector Ad-CMV-eGFP (10 ifu, 20 ifu, 50 ifu, 100 ifu, 200 ifu). Total RNA was extracted, and AT2R was detected by real time RT-PCR. The relative expression quantity (RQ) of AT2R following AT2R (10-200 ifu/cell) transduction is shown in Fig. 2a. Data indicate that Ad-G-AT2R-eGFP elicits AT2R overexpression in EJ cells, consistent with a previous report [23]. Similar results were obtained following Ad-G-AT2R-eGFP transduction of UM-UC-3 or 5637 cells (data not shown).

In addition, the fluorescence micrographs presented in Fig. 2b show that incubation of EJ cells with Ad-G-AT2R-eGFP (100 ifu/cell) produced a high level of expression of AT2R immunoreactivity and eGFP at 24 h after viral transduction when compared with EJ cells that had been infected with Ad-CMV-eGFP, which demonstrate only eGFP fluorescence.

### Effect of increased AT2R expression on cell proliferation in EJ cells

Analysis via a WST-1 assay of the proliferation of different types of BCa cells revealed that transduction with Ad-G-AT2R-eGFP (100 ifu/cell, 24 h) produced a significant cell growth inhibition when compared with cells transduced with Ad-CMV-eGFP (100 ifu/cell, 24 h) (Fig. 2c).

### AT2R overexpression induces apoptosis in bladder cancer cells

Transduction of BCa cells with Ad-G-AT2R-eGFP (100 ifu/cell) for 2 days resulted in a large number of cells that exhibited apoptotic-like morphologic characteristics, when compared with Ad-CMV-eGFP–treated EJ, UM-UC-3 and 5637 human BCa cells (Fig. 2d). The AT2R-expressing BCa cells exhibited irregular-shaped nuclei and a clear boundary between nuclei and cytoplasm when compared with the controls, consistent with our previous report using TUNEL labeling in AT2R-expressing DU145 cells [14]. The apoptotic action following AT2R transduction was confirmed by the finding that incubation of EJ cells with Ad-G-AT2R-eGFP (100 ifu/cell) for 2 days produced a significant increase in TUNEL labeling compared with the Ad-CMV-eGFP (100 ifu/cell)–treated cells (Fig. 2e and f).

### AT2R overexpression-induced changes in gene expression in EJ cells

**Fig. 3:**
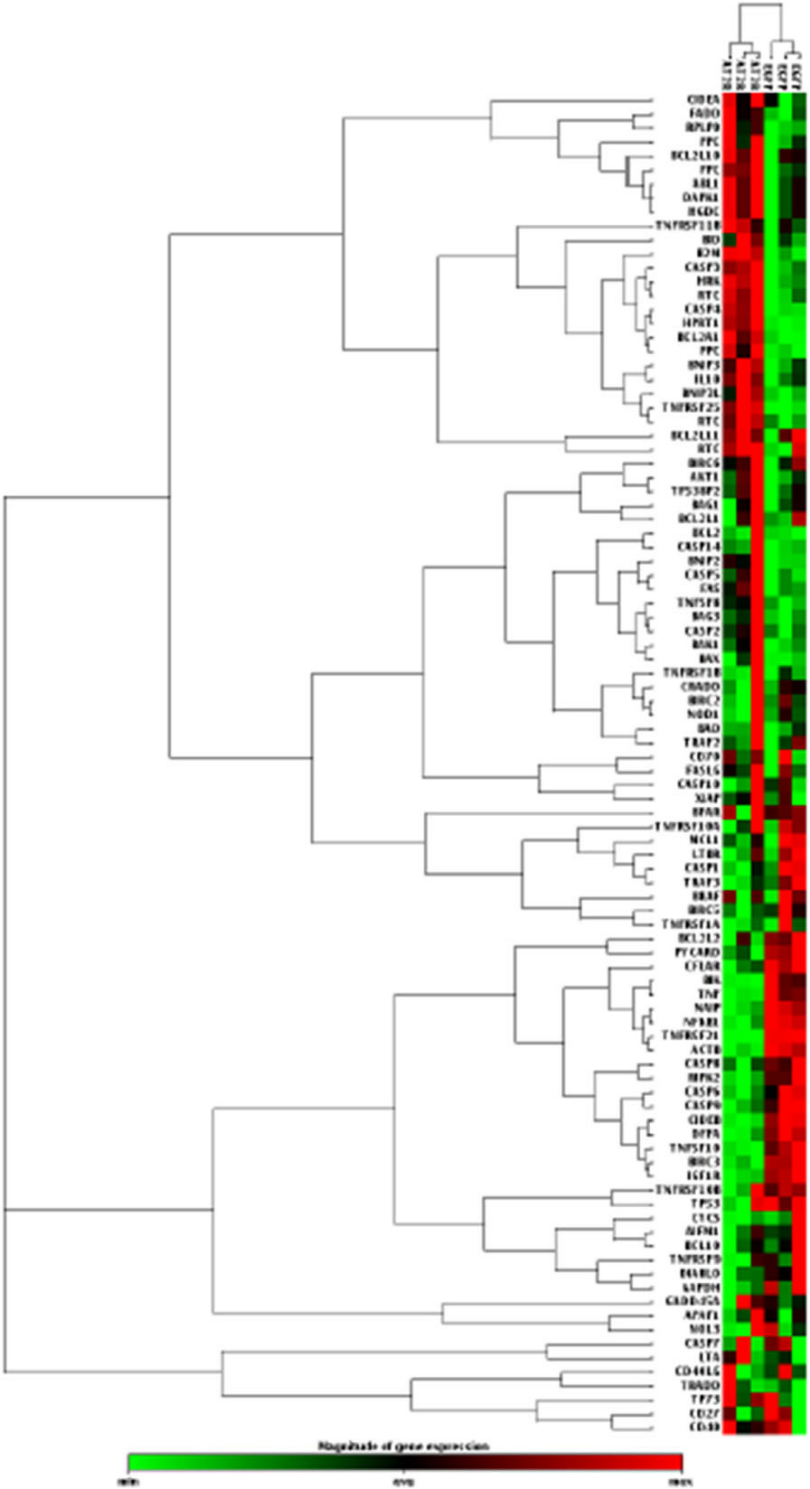
Cluster analysis of the up and downregulated genes in EJ cells transduced with Ad-G-AT2R-eGFP(100 ifu/cell). EJ cells were transduced with either Ad-CMV-eGFP or Ad-G-AT2R-eGFP (100 ifu/cell) for 24 h and then real-time PCR Array was prepared as described in Materials and Methods. Cells treated with Ad-CMV-eGFP (100ifu/cell) were used as a control

PCR Array analysis was performed to determine the molecular effects of AT2R expression in EJ cells. Of the 84 genes represented on the Human Apoptosis RT 2 Profiler PCR Array profiles, the expression levels of 2 genes (BCL2A1, TNFSF25) were upregulated and those of 9 genes (CASP 6, CASP 9, DFFA, IGF1R, PYCARD, TNF, TNFRSF21, TNFSF10, NAIP) were downregulated in EJ cells transduced with Ad-G-AT2R-eGFP (Table 2, Fig. 3). These differentially expressed genes can be allocated to genes encoding the tumor necrosis factor (TNF) ligand family (TNFSF10), the TNF receptor family (TNFRSF21, TNFRSF25), the Bcl-2 family (BCL2A1), the caspase family (CASP6, CASP9), the inhibitor of apoptosis proteins (IAP) family (NAIP), the caspase recruitment domain (CARD) family (PYCARD), as well as apoptosis regulators (Insulin-like growth factor 1 receptor-IGF1R and DNA fragmentation factor, 45 kDa, alpha polypeptide-DFFA).

**Table 1:** Primers for real-time RT-PCR

Gene	Sense	Sequence	Product size(bp)
Mas	Forward Reverse	5′- GCCTGTCAGTCCTTTACCCC-3′ 5′-CACAGAAGGGCACAGACCAA-3′	79
AT1R	Forward Reverse	5′-CCGCATTTAACTGCTCACACA -3′ 5′-ATCATGTAGTAGAGAACAGGAATTGCTT -3′	213
AT2R	Forward Reverse	5′-CGGAATTCATGAGCTGCGTTAATCC -3′ 5′-AACTGCAGTTAAGACACAAAGGTCTCCA -3′	165
Flt-1	Forward Reverse	5′- TCATGCTAATGGTGTCCCCG-3′ 5′-GTGCTGCTTCCTGGTCCTAAA-3′	99
Flk-1	Forward Reverse	5′-CAAGTGGCTAAGGGCATGGA -3′ 5′-ATTTCAAAGGGAGGCGAGCA-3′	181
VEGF	Forward Reverse	5′-AGGCCAGCACATAGGAGAGA-3′ 5′- TACCGGGATTTCTTGCGCTT-3′	141
GAPDH	Forward Reverse	5′-ACGGATTTGGTCGTATTGGG-3′ 5′-CGCTCCTGGAAGATGGTGAT-3′	214

**Table 2:** Differentially expressed genes in human BCa cells following Ad-G-AT2R-eGFP transduced

Name of gene	Description	Relative fold change (AT2R/eGFP)	Accession no. (Gene Bank ID)
BCL2A1	BCL2-related protein A1	8.57	NM_004049
CASP6	Caspase 6, apoptosis-related cysteine peptidase	0.38	NM_032992
CASP9	Caspase 9, apoptosis-related cysteine peptidase	0.44	NM_001229
DFFA	DNA fragmentation factor, 45 kDa, alpha polypeptide	0.46	NM_004401
IGF1R	Insulin-like growth factor 1 receptor	0.37	NM_000875
NAIP	NLR family, apoptosis inhibitory protein	0.44	NM_004536
PYCARD	PYD and CARD domain containing	0.41	NM_013258
TNF	Tumor necrosis factor	0.07	NM_000594
TNFRSF21	Tumor necrosis factor receptor superfamily, member 21	0.32	NM_014452
TNFRSF25	Tumor necrosis factor receptor superfamily, member 25	3.49	NM_003790
TNFSF10	Tumor necrosis factor (ligand) superfamily, member 10	0.20	NM_003810

### AT2R-induced apoptosis is mediated by Caspase-3

**Fig. 4:**
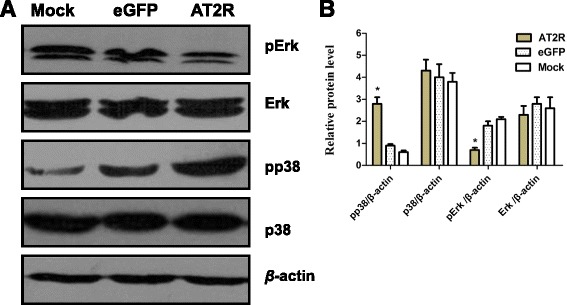
Involvement of MAPK superfamily in AT2R-induced apoptosis in BCa EJ cells. **a** representative Western blots show the p38 MAPK, pp38 MAPK, Erk and pErk in BCa EJ cells transduced for 24 h with Ad-G-AT2R-eGFP and Ad-CMV-eGFP (100 ifu/cell) or mock-transduced. **b** the relative values of MAPKs are presented as mean ± SD from three independent experiments. * *P* < 0.01

**Fig. 5:**
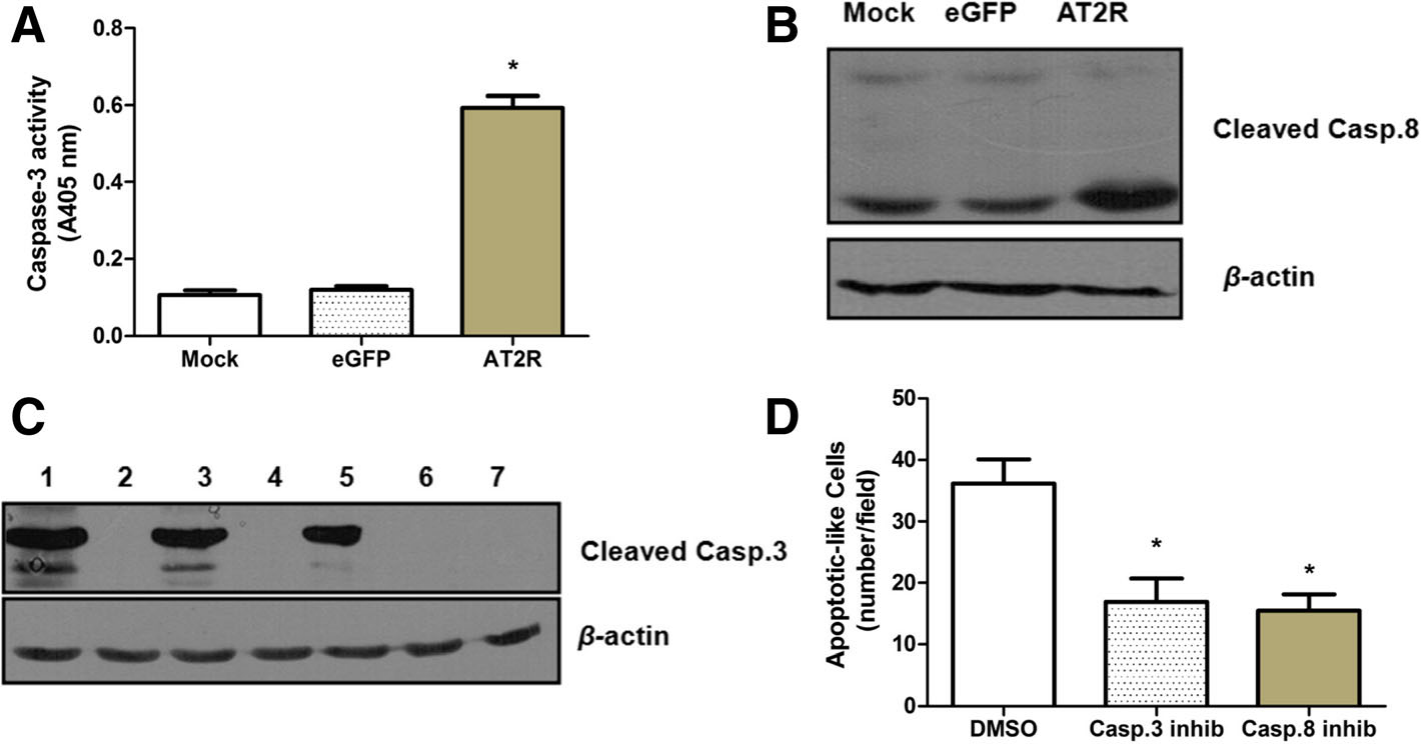
Role of caspases in AT2R-induced apoptosis in EJ bladder cancer cells. **a** AT2R-induced increase in caspase-3 activity in EJ cells. Cells underwent transduction with Ad-CMV-eGFP or Ad-G-AT2R-eGFP (100 ifu/cell) or were mock transduced for 2 d. Following this, cells were washed and lysed, and total cellular protein was assayed for caspase-3–like activity by measuring the release of pNA from the colorimetric caspase-3 substrate Ac-DEVD-pNA. Columns, mean A405 nm from three experiments; bars, SD. * *P* < 0.01 *vs* Ad-CMV-eGFP–transduced or mock-transduced cells. **b** Cells underwent transduction with Ad-CMV-eGFP or Ad-G-AT2R-eGFP (100 ifu/cell) or were mock transduced for 48 h. Western blot analysis for expression level of cleaved caspase-8 in cell lysates. **c** AT2R-induced increase in cleaved caspase-3 generation in EJ cells. Cells were infected with either 200, 100 or 50 ifu/cell of Ad-G-AT2R-eGFP (lanes 1, 3 and 5, respectively), 200, 100 or 50 ifu/cell of Ad-CMV-eGFP (lanes 2,4 and 6, respectively), or were mock transduced (lane 7). At 48 h after transduction, cell protein extracts were subjected to Western blot analysis. Representative blots indicating cleaved caspase-3 and *β*-actin under each treatment condition. **d** effects of caspase inhibitors on AT2R-mediated apoptosis in EJ cells. Cells were transduced with Ad-G-AT2R-eGFP (100 ifu/cell) for 6 h followed by treatment with either the caspase-3 inhibitor Ac-DEVD-CMK (20 μmol/L), the caspase-8 inhibitor Z-IETD-FMK (20 μmol/L) or control solvent (DMSO) for 24 h. *Green* fluorescent cells exhibiting apoptotic morphology were counted from 10 fields per well. *Columns*, mean of three experiments; bars, SD. * *P* < 0.01 *vs* control (DMSO-treated) cells

To understand the signaling pathway involved in AT2R medicated apoptosis, we measured the protein levels of MAPK superfamily proteins in EJ cells. Western blot analysis revealed increased levels of pp38 MAPK protein in the Ad-G-AT2R-eGFP-transduced cells when compared to the controls. Moreover, the Ad-G-AT2R-eGFP treatment decreased the levels of the anti-apoptotic protein p-ERK in the Ad-G-AT2R-eGFP transduced cells when compared to the controls (Fig. 4). In addition, transduction of EJ cells with Ad-G-AT2R-eGFP (100 ifu/cell) displayed significantly higher caspase-3–like activity compared with extracts from Ad-CMV-eGFP (100 ifu/cell)–transduced cells or mock-transduced cells (Fig. 5a). Further, EJ cells transduced with Ad-G-AT2R-eGFP (100 ifu/cell) resulted in cleavage of caspase-8 and consequently caspase-3 cleavage, and yielded the active subunits as shown by Western blotting analysis (Fig. 5b, c). No proteolytic processing of caspase-8 and caspase-3 was observed in control vector transduced or mock-transduced cells. Lastly, treatment of EJ cells with the caspase-3 inhibitor Ac-DEVD-CMK (20 μmol/L) significantly reduced the apoptosis elicited by transduction with 100 ifu/cell Ad-G-AT2R-eGFP (Fig. 5d). Interestingly, similar inhibition of AT2R-induced apoptosis was obtained by treatment of the EJ cells with the caspase-8 inhibitor Z-IETD-FMK (20 μmol/L) (Fig. 5d). Collectively, these data suggest the involvement of a caspase-8–mediated extrinsic signaling pathway followed by downstream activation of caspase-3 in the apoptosis elicited by overexpression of AT2R in bladder cancer cells.

### AT2R suppressed bladder tumor growth in nude mice

**Fig. 6:**
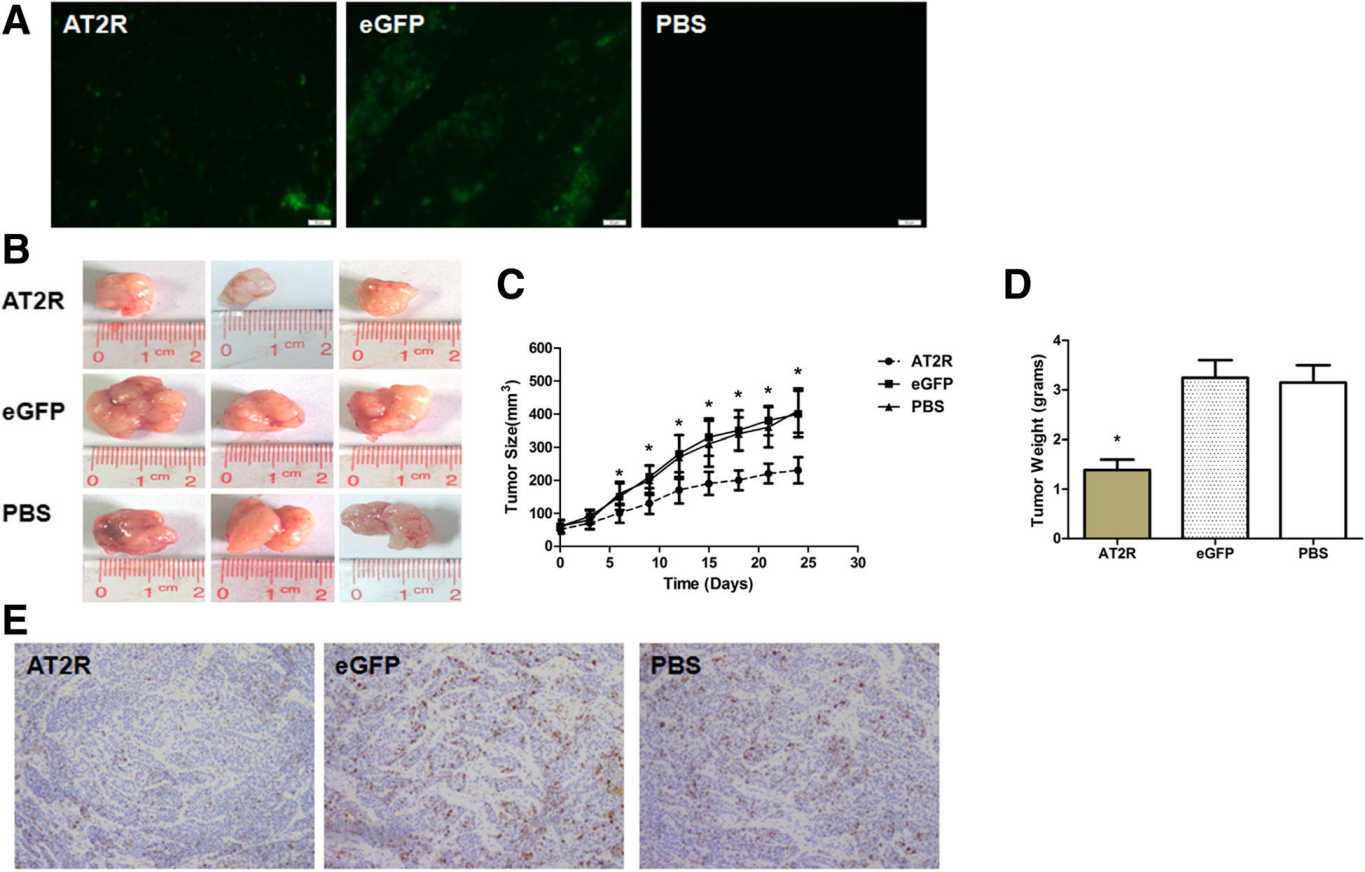
AT2R decreases nasopharyngeal tumor growth in nude mice. **a** Representative fluorescence micrographs (10× view) showing eGFP fluorescence from bladder tumor; **b** At the end of the experiment, mice were sacrificed to obtain the tumors. The size of human bladder tumor xenografts from mice injected with Ad-G-AT2R-eGFP, Ad-CMV-eGFP or PBS was measured using a caliper and volume was calculated as follows: volume = (D × d^2^)/2, where D is the longest diameter and d is the shortest diameter. *, *P* < 0.01; *n* = 6; **c** growth curve of tumor volumes; **d** tumors from mice treated with Ad-G-AT2R-eGFP, Ad-CMV-eGFP or PBS were weighed at the time of sacrifice. *, *P* < 0.01; *n* = 6; **e** sections of transplanted tumors infused with Ad-G-AT2R-eGFP, Ad-CMV-eGFP or PBS were stained with Ki-67. Representative photomicrographs are shown to the left (magnification × 200). * *P* < 0.01 vs. the control groups

EJ cells were injected subcutaneously into the dorsal flank of nude mice to assess the effect of AT2R on bladder tumor growth. When xenograft tumors were about to 50 mm^3^, mice were randomized for intratumor injections of Ad-G-AT2R-eGFP (1 × 10^9^ vg/mouse, 100 μl), Ad-CMV-eGFP (1 × 10^9^ vg/mouse, 100 μl) or PBS (100 μl) with multiple target points every 3 days to attain a high level of AT2R expression. Throughout this experiment, the mice were given 9 injections in total. The dose selection of the vector was based on preliminary experiments showing a high level of eGFP fluorescence within tumor sections (Fig. 6a). At this dose, there is no difference in body weight was found between the AT2R-treated and the control mice (data not shown). During the 25 days after the first Tumors injection, tumors in the two control groups of mice (PBS or Ad-CMV-eGFP-treated) continued to grew progressively (Fig. 6b, c), whereas administration of Ad-G-AT2R-eGFP resulted in a significant reduction in tumor volume. At the end of the experiment, tumors from mice in the two control groups were >3-fold larger than their initial size, and were about double the size of tumors from the Ad-G-AT2R-eGFP-treated mice. The mice were euthanized at the end of the study and the tumors were dissected and weighed. As shown in Fig. 6d, the tumors from mice treated with Ad-G-AT2R-eGFP weighed about 50% less than the tumors from mice infused with Ad-CMV-eGFP and PBS, demonstrating that AT2R reduces tumor growth.

### AT2R reduces cell proliferation in EJ xenograft tumors

Tumor sections from mice injected with viral vectors or PBS underwent immunostaining using antibodies to the Ki67 markers of cell proliferation. The results showed that both the staining intensity and the number of hyper-proliferative Ki-67 tumor cells were significantly decreased in the Ad-G-AT2R-eGFP-treated group compared with both control groups (Fig. 6e), suggesting that AT2R reduces cell proliferation in vivo.

### AT2R induced apoptosis in vivo

**Fig. 7:**
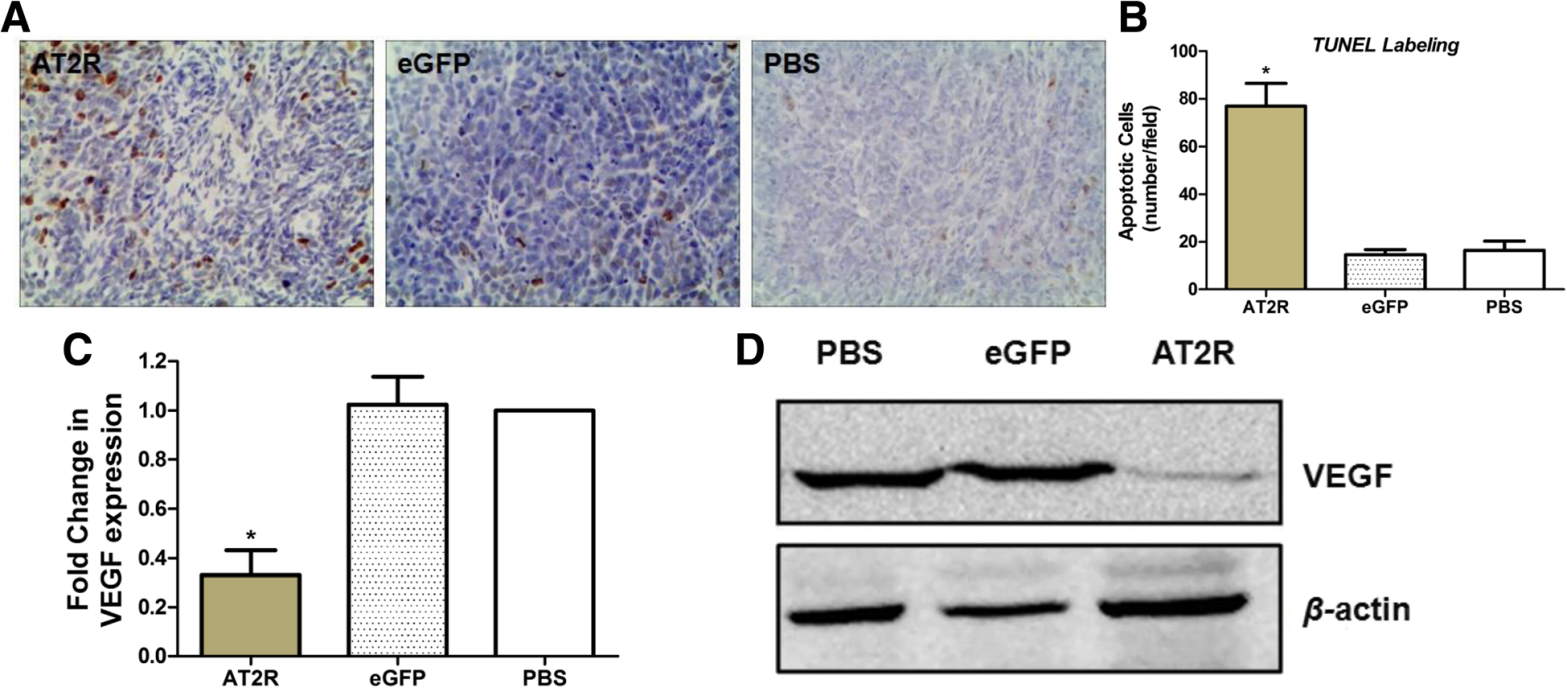
Effect of AT2R on apoptosis and VEGF expression in bladder tumor xenografts. **a** apoptosis in tumor sections from bladder tumor xenografts was identified by DeadEnd Colorimetric TUNEL System kit. Two representative phase-contrast micrographs from each treatment condition. The brown-colored nuclei represent TUNEL-positive (apoptotic) cells; **b** TUNEL-positive cells were quantified as the average of 5 fields selected per tumor (magnification × 200); **c** levels of VEGF in tumor tissue from bladder tumor xenografts were measured by real-time RT-PCR; **d** VEGF protein expression was detected by Western blot. Columns, mean from 6 separate experiments; bars, SD. * *P* <0.01 vs. the control groups

Apoptosis was detected in bladder cancer xenograft tumors to determine whether AT2R reduces angiogenesis. Apoptotic cells were identified by positive immunoreactivity to TUNEL. A marked increase in immunoreactive TUNEL-stained cells was observed in tumor tissue sections from mice treated with Ad-G-AT2R-eGFP (75 ± 4.7/field) as compared to mice treated with Ad-CMV-eGFP (14 ± 3.1/field) or PBS (20 ± 3.6/field) (Fig. 7a, b), suggesting that AT2R significantly induced cell apoptosis in xenograft bladder tumors.

### Effect of AT2R on VEGF in bladder tumor xenografts

It was reported that AT2R was associated with increased VEGF secretion at low Ang-II concentrations [25] and our in vivo study also found that moderately increasing AT2R expression could increase the growth of HCC tumors [18]. To characterize these effects in this experiment, we analyzed the VEGF expression level from the xenograft tumors. Our results shown that VEGF mRNA and protein were significantly reduced in tumors from nude mice administered Ad-G-AT2R-eGFP when compared to tumors from Ad-CMV-eGFP or PBS treated animals (Fig. 7c, d).

## Discussion

In this study, we investigated the effects of adenoviral mediated overexpression of AT2R on BCa cells and xenograft tumor models of BCa. Overexpression of AT2R by Ad-G-AT2R-eGFP transduction significantly reduced the viability of BCa cells and promoted apoptosis. The induction of apoptosis is associated with activation of p38 MAPK and inactivation of ERK MAPK, and is partly dependent upon activation of caspase-8 and caspase-3. Furthermore, the data also indicated that overexpression of AT2R significantly reduces human bladder xenograft growth and tumor angiogenesis.

We first examined the expression of AT1R, AT2R, and Mas in clinical tissue samples of bladder carcinoma. It is widely acknowledged that, apart from AT1R, RAS harbors other receptor subtypes, some of those which mediate actions opposing those of AT1R [26]. AT2R and Mas are the most well characterize of these opposing or protective receptors with the Mas-related G-protein-coupled receptor (MrgD) as a more recently discovered candidate [27]. Most importantly, AT2R and Mas mediate a multitude of strikingly similar tissue protective and regenerative effects including anti-inflammatory, anti-fibrotic, neuroregenerative, vasodilatory, and beneficial metabolic effects [28–30].

Our results indicate that AT1R was upregulated and AT2R was downregulated in bladder carcinoma tissues (Fig. 1). Consistent with our results, a recent study demonstrated that AT1R was upregulated in human bladder cancer and greater expression of AT1R was associated with greater microvessel density [31]. There is also a direct correlation between AT1R expression and tumor stage and liver metastasis and corresponding inverse correlation with AT2R expression in human colorectal cancer [25].

In our study, Mas was also significantly upregulated in human bladder tissues (Fig. 1), consistent with our previous study showing that Mas was upregulated in human nasopharyngeal carcinoma tissues [32]. Recently, it has also been demonstrated that Mas is increased in colonic adenocarcinomas [33] and higher expression of Mas is observed in hepatic colorectal metastases when compared to the surrounding liver tissue [34]. To our knowledge, this is the first time that expression levels of AT1R, AT2R and Mas have been assessed in human bladder tissue and our results suggest that AT1R, AT2R, and Mas can be therapeutic targets.

Various sources have demonstrated AT2R overexpression-induced apoptosis in a variety of cell lines [15–19]. Apoptosis is an important mechanism by which cancer therapeutic agents can induce cancer cell death. Here, we show that endogenous AT2R mRNA levels were below the detection limit and Ad-G-AT2R-eGFP induced overexpression of AT2R in all three BCa cell lines produced apoptosis. In a recent study, we have demonstrated AT2R induced prostate cancer cell apoptosis in prostate xenograft tumors [35]. However, our previous study also showed that high levels of AT2R elevated apoptosis in HCC cell lines, while moderate and low level of AT2R did not impact apoptosis [18]. Due to the highly sensitive nature of this AT2R mediated effect, it is important to obtain a more comprehensive and deeper understanding of the signaling and mechanisms underlying AT2R activation.

AT2R-mediated apoptosis undergoes different cellular mechanisms of apoptosis depending on the cell type. In a rat insulinoma cell line such as the INS-1O, for example, overexpression of AT2R induces caspase-8, caspase-9, and caspase-3 cleavage and decreases Bcl-2, pAkt, and pERK expression levels [36]. In intestinal epithelial cells, Ang II signals through AT2R to upregulate GATA-6 expression which in turn upregulates the expression of Bax and eventually leading to apoptosis in these cells [15]. In HL-1 cardiomyocytes, iNOS upregulation following induced AT2R expression seems to be the basis for increased in cardiomyocyte apoptosis [16]. In another case, AT2R signaling stimulates the MAPK tyrosine phosphatase, (MKP)-1, which inhibits MAPK activation and consequently inactivates Bcl-2 and induces apoptosis in proximal tubular cells [37]. Our previous studies suggest that AT2R-mediated apoptosis was mediated by p38 MAPK and caspase-3 and downregulation of Gadd45a, TRAIL-R2, and harakiri Bcl-2-interacting protein (HRK) in prostate cancer cells [17, 23]. In HCC cell lines, we observed activation of p38 MAPK and phosphorylated c-Jun N-terminal kinase (pJNK) [18]. Consistent with the latter, the current experiments indicate that AT2R overexpression induced apoptosis in BCa cells is mediated via an extrinsic cell death signaling pathway that is dependent on activation of p38 MAPK, caspase-8, and caspase-3 and downregulation of Erk/MAPK.

To our knowledge, this is the first study which clearly assesses apoptosis-related gene expression profiles associated with AT2R-induced apoptosis in BCa cells. We observed that forced overexpression of AT2R resulted in significant changes in a large number of genes including both pro-apoptotic genes and anti-apoptotic genes, as determined by PCR array analysis. BCL2A1, which was elevated to 8.57-fold above control values, belongs to the pro-survival BCL2 family and is one of the less extensively studied anti-apoptotic proteins. BCL2A1 has been recently described as an oncogene responsible for resistance to BRAF inhibition in melanoma [38]. A study also showed that elevated BCL2A1 protein prevents apoptosis [39]. In the present study, BCL2A1 was upregulated, suggesting that BCL2A1 may actually oppose the apoptotic effect that is induced by AT2R in BCa.

Moving on to TNF, TNFα is a double-edged sword in tumor development. In most cases, TNFα acts as a promoter rather than a killer in tumor cells and tissues [40]. It should be noted that in our case the expression of TNF was 0.07-fold of control values. This leads to the suspicion that TNF may be a negative regulator in AT2R induced apoptosis in BCa cells. Our results showed that the expression of TNFRSF25 (DR3) was increased, while TNFSF10 (TRAIL) and TNFRSF21 (DR6) were downregulated. Interestingly, TNFSF10 was also downregulated and TNFRSF10B (DR5) was increased in AT2R-mediated apoptosis in prostate cancer cells in our previous study [23]. In addition, others have demonstrated that several cancers are resistant to TRAIL-induced cytotoxicity and increased expression of the DR could overcome human cancer resistance to TRAIL [41, 42]. Overall, it can be said that other factors or other mechanisms may be important regulators of sensitivity to TRAIL-induced apoptosis in these cancer cells. Here, we would like to conclude that TNFSF10 (TRAIL) and DRs were involved in the AT2R induced apoptosis in BCa cells, however the mechanisms or the sensitivity to TRAIL-induced apoptosis in BCa needs to be studied further.

Apoptosis-associated genes like DFFA, PYCARD, NAIP, IGF1R, CASP6 and CASP9 were also up or downregulated in the present study. However, these genes have not been observed to change in our previous study in AT2R-expressing DU145 cells. In all, these results may help to elucidate the complicated mechanisms of AT2R inducing apoptosis in different tissues and cells.

Angiogenesis is a complex process and a large number of factors are involved in tumor angiogenesis. A recent study notes an AT1R antagonist as an angiogenic inhibitor in a xenograft model of bladder cancer [12] and prostate cancer [43]. There is also evidence showing that AT1R was associated with colorectal tumor VEGF-A secretion and microvessel density of bladder cancer [25, 31], suggesting that AT1R could regulate tumor angiogenesis. Kosugi et al demonstrated that an AT1R antagonist could prevent tumor growth and angiogenesis in xenograft models of human BCa using KU19-19 cells through the suppression of VEGF [12, 13]. In most pathophysiological situations and in some experimental models of angiogenesis [44], AT2R is thought to oppose the actions of the AT1R subtype. Yet some data indicate that AT2R may be pro-angiogenic [45] and works in concert with the AT1R subtype to increase VEGF levels and blood vessel formation [46, 47]. These conflicting reports leave open the question of whether AT2R activation has beneficial or deleterious effects on tumor angiogenesis. In our case, we found that AT2R markedly reduced VEGF expression in human bladder tumor xenografts, suggesting that this growth factor is involved in the antiangiogenic response to the receptor. A significant decrease of VEGF mRNA was observed in the tumors from mice treated with Ad-G-AT2R-eGFP when compared with tumors from control animals. VEGF receptors (Flt-1 and Flk-1) were also reduced in the tumors from mice treated with Ad-G-AT2R-eGFP when compared with the controls (Fig. 7). Taken together, these results demonstrate that VEGF downregulation is also responsible for the observed AT2R-mediated reduction in human bladder xenograft growth and tumor angiogenesis.

## Conclusions

In summary, we have shown that intratumor administration of adenovirus-based therapeutic gene delivery causes high gene expression in vivo. The administration of Ad-G-AT2R-eGFP then significantly attenuates the growth of bladder carcinoma tumors suggesting the effectiveness of this adenovirus-based gene therapy for bladder cancer treatment. The underlying mechanism for the reduced human bladder xenograft growth and tumor angiogenesis as suggested by the results is AT2R inhibiting proliferation and promoting cell apoptosis of human BCa cells. In all, these data suggest that the AT2R may serve as a novel anti-angiogenic treatment for BCa and a potential target gene for bladder carcinoma gene therapy.
